# Monosodium Glutamate Inhibits *Pseudomonas aeruginosa*-Induced Acute Lung Injury by Targeting the Type III Secretion Systems and Modulating Host Immunity

**DOI:** 10.3390/microorganisms14030725

**Published:** 2026-03-23

**Authors:** Jing Xu, Weiwei Wang, Yaxin Zhou, Hongxing Zhang, Zixuan Shang, Zhijin Zhang, Bing Li, Yubin Bai, Jiyu Zhang

**Affiliations:** 1 Key Laboratory of New Animal Drug Project of Gansu Province, Key Laboratory of Veterinary Pharmaceutical Development of the Ministry of Agriculture and Rural Affairs, Lanzhou Institute of Husbandry and Pharmaceutical Sciences, Chinese Academy of Agricultural Sciences, Lanzhou 730050, China; 2 New Veterinary Drugs in Gansu Province Engineering Research Center, Key Laboratory of New Veterinary Drugs Engineering, Laboratory Animal Science Data Center of Gansu Province, Lanzhou Institute of Husbandry and Pharmaceutical Sciences, Chinese Academy of Agricultural Sciences, Lanzhou 730050, China

**Keywords:** monosodium glutamate, acute lung injury, type III secretion systems, PopB

## Abstract

The opportunistic pathogen *Pseudomonas aeruginosa* poses a serious threat to immunocompromised patients. Monosodium glutamate (MSG), a widely used flavor enhancer, has been reported to possess anti-inflammatory and antioxidant properties. However, its therapeutic potential and mechanism against *Pseudomonas aeruginosa* (*P. aeruginosa*) infection have remained unexplored. This study systematically elucidated the protective effects and molecular mechanisms of MSG against *P. aeruginosa*-induced acute lung injury (ALI). In a murine pneumonia model, MSG administration effectively alleviated lung pathological damage, edema, and inflammatory responses. Mechanistically, MSG exerted protection through a multifaceted strategy, including direct suppression of bacterial virulence via binding to PopB of T3SS inhibition of the TLR4/MyD88/MAPK-driven inflammatory cascade and pro-inflammatory cytokine production, enhancement of endogenous antioxidant defense (SOD, CAT), and reshaping of pulmonary macrophages from the M1 to M2 phenotype. Notably, the anti-virulence effect of MSG, achieved by binding to PopB (K_D_ = 3.52 × 10^−6^ M), presented a distinct advantage over traditional antimicrobials by potentially mitigating resistance development. Collectively, these findings indicated that MSG can alleviate ALI caused by *P. aeruginosa* infection.

## 1. Introduction

*Pseudomonas aeruginosa* (*P. aeruginosa*) is an opportunistic zoonotic pathogen widely present in the environment and possesses three important virulence systems: the quorum sensing system, the biofilm system, and the secretion system [1]. The current mainstream treatment strategy for bacterial infections remains the use of antibiotics; however, the prolonged and widespread use of antibiotics, coupled with their inappropriate administration and even unregulated misuse, has led to a sharp increase in antibiotic resistance [2]. Ranking among the WHO’s most drug-resistant bacteria, *P. aeruginosa* is a major cause of nosocomial infections, accounting for over 10% of cases, including pneumonia and bloodstream infections [3,4]. Hospital-acquired pneumonia (HAP) currently accounts for an extremely high proportion of hospital-acquired infections, reaching as high as 80%, making it one of the most prevalent types of healthcare-associated infections [5,6,7]. *P. aeruginosa* causes difficult-to-treat acute and chronic pulmonary infections, with treatment challenges stemming from its inherent resistance, biofilm formation, and adaptability [8]. Therefore, preventing HAP is a top priority in hospital infection control efforts [9]. Consequently, developing novel therapeutic agents, especially those targeting key virulence factors or resistance mechanisms, to effectively combat its multidrug resistance is an urgent priority. Thus, overcoming *P. aeruginosa* infections requires a new multi-pronged strategy. Exploring innovative therapies that act beyond traditional antibiotic targets and effectively intervene in the infection process has become a current focus of research.

In pneumonia, *P. aeruginosa* causes tissue damage and declining lung function by triggering an excessive inflammatory response [10]. The malignant deterioration of lung function is perpetuated by a vicious inflammatory cycle involving barrier breakdown, resolution failure, and fibrotic remodeling [11,12]. In acute *P. aeruginosa* infection, the dynamic conversion of macrophages from the pro-inflammatory M1 to the reparative M2 phenotype is a pivotal regulatory step for controlling inflammation and pathology [13,14]. As a characteristic and critical virulence factor in acute *P. aeruginosa* pneumonia, the type III secretion system injects effectors into host cells to modulate signaling and induce cellular damage [15]. Therapeutically targeting the T3SS can reduce virulence without imposing survival stress, thereby lowering resistance risk [16,17]. Given this, the development of small-molecule inhibitors targeting T3SS and immunomodulatory strategies holds promise as a novel therapeutic approach for treating *P. aeruginosa* infections, particularly against multidrug-resistant strains. Such interventions not only help mitigate excessive immune responses triggered by infection but also offer a promising therapeutic strategy for reducing antibiotic selection pressure and delaying the evolution of drug resistance.

Monosodium glutamate (MSG) is a classic experimental inducer used to model hypothalamic injury-induced obesity, particularly when studying central nervous system dysfunction-driven metabolic syndromes characterized by severe insulin resistance and visceral adiposity [18,19,20]. However, the MSG dosage used in scientific research and modeling is significantly higher than normal daily dietary intake in humans; therefore, it is valuable to examine the potential positive effects of MSG, such as its observed association with a reduced risk of hyperglycemia in Chinese adults [21]. As a flavor enhancer that is metabolized into physiologically active glutamic acid in the body, MSG can provide both gustatory and potential nutritional benefits when used appropriately. The World Health Organization (WHO), the U.S. Food and Drug Administration (FDA), and the Scientific Committee on Food (SCF) of the European Union, among other renowned institutions, have all endorsed monosodium glutamate as a safe and reliable food additive [22]. Studies have shown that MSG can promote energy storage via mechanisms that include enhanced insulin sensitivity and inhibition of lipolysis [23]. Animal studies have indicated that appropriately dosed MSG, especially in combination with L-arginine, can enhance energy metabolism and antioxidant capacity by improving feed efficiency and lipid metabolism [24]. However, despite these recognized metabolic benefits, it is unclear whether MSG can exert similar defensive or restorative effects in the context of an infectious challenge. Given that *P. aeruginosa* pneumonia disrupts host energy balance and induces oxidative damage, this study explored whether MSG, acting as an immunometabolic modulator, could support host homeostasis to combat infection and inflammation in a pneumonia model.

Excitingly, this study reveals that MSG significantly reduces pulmonary bacterial burden and alleviates histological damage in a murine model of acute *P. aeruginosa* pneumonia, while concurrently exhibiting distinct immunomodulatory properties. More significantly, these discoveries provide potentially novel molecular entities for the creation of novel synergistic antibacterial strategies derived from food components, as well as a crucial basis for their subsequent transformation from well-known food components to anti-infective agents with immunomodulatory functions.

## 2. Materials and Methods

### 2.1. Materials

Monosodium glutamate (purity 99.23%; Cat#: HY-W250122) was purchased from MedChemExpress (MCE, Shanghai, China). The mouse alveolar epithelial cells (MLE-12; Cat#: STCC20008), mouse alveolar macrophage cell line (MH-S; Cat#: GNM43), and Chinese hamster ovary epithelial cell line (CHO-K1; Cat#: GNHa7) were all purchased from the Cell Bank/Stem Cell Bank, Chinese Academy of Sciences (Shanghai, China). DMEM (Cat#: C11990055BT), F-12K medium (Cat#: 21127030), RPMI 1640 medium (Cat#: 11875101), penicillin/streptomycin (Cat#: 15140122), 0.25% trypsin-EDTA solution (Cat#: 15050065), fetal bovine serum (Cat#: FBP-C520), and LiveBLAzer™ FRET-B/G Loading Kit (containing CCF4-AM) were all purchased from Thermo Fisher Scientific (Shanghai, China) Co., Ltd. (Shanghai, China). Cell Counting Kit-8 (CCK-8; (Cat#: HY-K0301) was also purchased from MedChemExpress (MCE, Shanghai, China). LB liquid medium (Guangdong Huankai Microbial Sci.&Tech. Co., Ltd., Guangzhou, China; Cat#: 028320) and LB agar media (Guangdong Huankai Microbial Sci.&Tech. Co., Ltd., Guangzhou, China; Cat#: 028330) were formulated in accordance with the product specifications and the appropriate proportion of water. PME6032 vector (Cat#: ZCF077C) was synthesized in Sangon Biotech (Shanghai, China). Isoflurane (Cat#: R510-22-10) was purchased from RWD Life Science (Shenzhen, China). Ampicillin (Cat#: HY-B0522), 0.22 μm filter (Cat#: 230922-051-A), and DH5α competent cells (Cat#: 9057) were purchased from Takara Bio (Beijing, China). The LDH kit (A020-2-2) was purchased from Nanjing Jiancheng Bioengineering Institute (Nanjing, China). The antibodies for phospho-NF-κB p65 (82335-1-RR), p-65 (80979-1-RR), phospho-P38 (14064-1-AP), phospho-JNK (80024-1-RR), MyD88 (23230-1-AP), TLR-4 (19811-1-AP), P38 (14064-1-AP), JNK (51153-1-AP), and β-actin (66009-1-Ig) were purchased from ProteinTech Group (Wuhan, China). The ELISA kits for IL-1β (Catalog No. JL18442-96T), MPO (Catalog No. JL10367-96T), SOD (Catalog No. JL12237-96T), and CAT (Catalog No. JL18163-96T) were all purchased from Jianglai Biotechnology (Shanghai, China).

### 2.2. Animal Experiment

A total of 60 SPF-grade healthy male C57BL/6J mice (6–8 weeks old, body weight approximately 20 g) were provided by Lanzhou Veterinary Research Institute of Chinese Academy of Agricultural Sciences (CAAS), Lanzhou, China. All mice were housed in the specific pathogen-free (SPF) facility of the DawaShan Animal Facility, Lanzhou Institute of Husbandry and Pharmaceutical Sciences of CAAS. During the study, the mice were allowed free access to food and water and were maintained on a 12 h light/dark cycle (lights on from 8:00 to 20:00). Following a week of acclimatization, 60 mice were randomly divided into five groups (n = 12 per group) by GraphPad Prism: the normal group (Normal), the model group (PAO1 infected group) (Model), the MSG low-dose group (50 mg/kg), the MSG medium-dose group (100 mg/kg), and the MSG high-dose group (200 mg/kg).

The animal study protocol was approved by the Animal Ethics Committee of Lanzhou Institute of Husbandry and Pharmaceutical Sciences of CAAS (Approval No. 2025-53). To minimize potential bias, all outcome assessments—including histological evaluation, biomarker measurement, and image analysis—were performed by investigators blinded to the group allocation. Drug administration and sample collection were conducted separately. Statistical analysis of the data was performed only after completion of all the experimental stages and removal of blinding. And all animal experimental procedures were conducted in accordance with the relevant guidelines and regulations of the Animal Ethics Committee of the Lanzhou Institute of Husbandry and Pharmaceutical Sciences of CAAS.

### 2.3. Construction of a PAO1-Induced Acute Lung Injury (ALI) Model

A total of 24 SPF-grade healthy male C57BL/6J mice (6–8 weeks old, body weight approximately 20 g) were provided by the Lanzhou Veterinary Research Institute of CAAS (Lanzhou, China). All mice were housed in the specific pathogen-free (SPF) facility of the DawaShan Animal Facility, Lanzhou Institute of Husbandry and Pharmaceutical Sciences of CAAS. During the study, mice were allowed free access to food and water and were maintained on a 12 h light/dark cycle (lights on from 8:00 to 20:00). Following a week of acclimatization, 24 mice were randomly divided into five groups (n = 12 per group) by GraphPad Prism: the model group (Model; PAO1 infected group) and the control group (Control). The model group was created by administering 50 μL of 4 × 10^6^ PAO1 colony-forming units (CFU) in the endotracheal tube. As a vehicle control, 50 μL of sterile saline was administered endotracheally to the control group. Twenty-four hours after model establishment, all mice were humanely euthanized via respiratory anesthesia with isoflurane. Immediately following euthanasia, the trachea was cannulated, and bronchoalveolar lavage was performed twice using 0.5 mL of sterile saline per lavage. The recovery rate of the lavage fluid exceeded 60%. Subsequently, both bronchoalveolar lavage fluid (BALF) and lung tissue samples were immediately snap-frozen in liquid nitrogen and stored at −80 °C until further analysis.

### 2.4. Cell Culture

Dulbecco’s modified Eagle’s medium (DMEM) with 10% fetal bovine serum (FBS) and 1% penicillin/streptomycin was used to cultivate MLE-12 cells in T75 flasks at 37 °C with 5% CO_2_. Similarly, 10% FBS and 1% penicillin/streptomycin were added to RPMI-1640 medium and F-12K medium, respectively, to cultivate MH-S and CHO-K1 cell lines. Fresh medium was replenished every 2–3 days, and cells were passaged when reaching 80–90% confluence. After detaching the cells using a 0.25% trypsin-EDTA solution at 37 °C for 2–3 min, the cells were neutralized with complete media that contained serum. After that, the cell suspension was centrifuged for 5 min at 1000 rpm. Following centrifugation, the cell pellet was resuspended in fresh complete medium and sub-cultured at a split ratio of 1:3.

### 2.5. Cell Viability Assay

The safe drug concentration of MSG was determined by using CCK8. The cells were seeded into 96-well plates at a density of 5 × 10^3^ cells per well. After 24 h, the cells were completely adherent to the wall, and the original complete medium was removed. Then, DMEM, F-12K, or RPMI-1640 containing various concentrations of MSG were added for culture. After 12 h of culture, the medium containing the drug in each well was removed, and 100 μL of basic medium (DMEM, F-12K, or RPMI-1640) containing 10% CCK-8 reagent was added to each well. After that, the 96-well plates were kept in the incubator with 5% CO_2_ at 37 °C for 1 h to 4 h. Following the detection of each well’s absorbance value at 450 nm, the cell survival rate was computed using the formula:

(1) $$\mathrm{Cell}\;\mathrm{viability}\;(\%)\;=\;\frac{{OD}_{treatment\;}\;-\;{OD}_{blank\;}}{{OD}_{control\;}\;-\;{OD}_{blank\;}}\times \;\mathrm{100\%}$$

where the treatment was the well with the medium containing the drug and the cells, blank was the well with only the basic medium but no cells, and control was the well with the cells and only the basic medium. Each treatment group contained 6 replicates.

### 2.6. Bacterial Preparation

*P. aeruginosa* (PAO1 and PA14) were retrieved from the −80 °C freezer in the laboratory and reactivated in LB liquid medium. To help the bacteria recover from dormancy and start the active growth phase, they were cultured for 12–16 h at 37 °C at 100 rpm. The bacteria were subsequently sub-cultured twice in sterile LB medium to verify their existence in the exponential growth phase, demonstrating a stable condition and normal virulence expression. In each bacterial infection experiment, the OD_600_ value was calibrated to 0.7 using the cell density meter (WPA CO8000, Biochrom, Cambridge, UK). The standardized bacterial solution was employed to infect the well-cultured MLE-12, MH-S, and CHO-K1 cells.

### 2.7. Establishment of the Cell Infection Model

This work established three cell infection models: MLE-12, MH-S, and CHO-K1. All cells were consistently grown in the appropriate complete medium, including 10% fetal bovine serum and 1% penicillin-streptomycin. Before beginning the infection experiment, cells in the logarithmic growth phase were injected into 96-well plates at a density of 5.0 × 10^3^ per well. Following the cells becoming fully attached, the pathogens were sequentially introduced. MLE-12 cells: the standard strain PAO1 of *P. aeruginosa* was inoculated at a multiplicity of infection (MOI) of 10, with samples collected 3 h post-infection; MH-S cells: PAO1 was inoculated at an MOI of 10, with samples collected 1.5 h post-infection; and CHO-K1 cells: the *P. aeruginosa* strain PA14 was inoculated at an MOI of 10, with samples collected 1 h post-infection. All samples after the infection were promptly processed for subsequent RT-qPCR or immunofluorescence detection and analysis.

### 2.8. Establishment and Verification of Model Strains

The PME-exoT-bla vector was produced by utilizing the PME6032 vector, incorporating the complete PAO1 effector *exoT* gene sequence together with the full-length *bla* fusion gene sequence, followed by amplification of the plasmid. The plasmid was electroporated into the wild-type PAO1 competent cells, plated on LB agar media supplemented with 50 μg/mL of ampicillin, and after 24 h of incubation, a single colony was selected and cultured in LB liquid medium containing ampicillin.

Then, using PAO1 competent cells containing the PME-exoT-bla vector, this was infected with MLE-12, and Fluorescence Resonance Energy Transfer (FRET) technology was used to quantitatively calculate β-lactamase activity or the inhibition efficiency of corresponding inhibitors. The cultured single colony was preserved at −80 °C with 50% glycerol for long-term storage.

### 2.9. Histopathological Examination of Mice Lung Tissues

First, the lung tissue fixed in 4% paraformaldehyde was prepared into wax blocks, followed by hematoxylin and eosin (HE) staining. After fixation, gradient dehydration was performed, immersed in xylene, and then paraffin embedding was performed. After sectioning with a microtome and baking and once firmly adhered to the slide, the tissue was immersed in xylene to remove the paraffin, then sequentially immersed in graded ethanol (100%, 95%, 80%, 70%), and finally rinsed with distilled water. After staining with hematoxylin solution, the excess stain was removed. The sections were then immersed in 1% hydrochloric acid ethanol until the nuclei became clearly visible. After immediately rinsing the sections and immersing them in a weak alkaline solution for blueing, this was followed by rinsing with water. Subsequently, the sections were immersed in eosin solution for staining, the excess stain was removed and then dehydrated using an ethanol gradient. Then, the sections were immersed in xylene for clearing twice. Finally, a neutral resin was applied to the sections and covered with a coverslip while avoiding bubbles, and air-dried at room temperature. The sections were examined under an optical microscope, and three fields of view per section were randomly selected for evaluation and measurement of the thickness of pulmonary septa to assess the degree of lung tissue damage.

### 2.10. Immunofluorescence

The expression levels of CD86 and CD206 in lung tissue were analyzed using an immunofluorescence staining kit (Servicebio Technology; Cat#: G1226, Wuhan, China). In brief, lung tissue sections underwent sectioning, antigen retrieval, and blocking with 5% bovine serum albumin (BSA). Subsequently, tissue sections were incubated separately with anti-CD86 (1:2000) or anti-CD206 (1:2000) antibodies at 4 °C, followed by incubation with enzyme-labeled goat anti-rabbit IgG at room temperature for 1 h. After being washed with PBS, the slides were incubated for 10 min with iFluor488 or iFluor555 amide working solutions targeting CD86 and CD206, respectively. Following DAPI staining and washing, coverslips were mounted, and images were acquired using a Pannoramic MIDI (3D HISTECH, Budapest, Hungary) at 400× magnification.

MH-S cells were seeded at 1.5 × 10^5^ cells/well in 24-well plates, pretreated with MSG (100 μM) for 1.5 h, then infected with PAO1 at an MOI of 10 for 1.5 h, and fixed in 4% paraformaldehyde for 15 min. After 15 min penetration with 0.1% Triton X-100, cells were blocked with 5% BSA for 30 min, then incubated overnight at 4 °C with rabbit anti-CD86 (1:50) or anti-CD206 (1:200) antibody probes, and followed by incubation at room temperature in the dark with FITC- or DyLight550-conjugated goat anti-rabbit IgG (H+L) secondary antibodies. Finally, cells were stained with DAPI and imaged at 400× magnification using a Pannoramic MIDI scanner (3D HISTECH, Budapest, Hungary).

### 2.11. Quantitative Reverse Transcription-Quantitative Polymerase Chain Reaction (RT-qPCR)

Total RNA was extracted from tissues using the SteadyPure Universal RNA Extraction Kit (AG Company (Shanghai, China); cat:123). Total RNA was then reverse transcribed into cDNA using the Reverse Transcription Reagent Premix (TCH026; Takara). Subsequently, the cDNA obtained from the reverse transcription step was used as a template. SYBR Green dye (cat:RR037A; Takara) was employed to detect mRNA expression on the RT-qPCR instrument. For data analysis, β-actin was used as the internal reference gene. The relative expression levels of each mRNA were calculated using the comparative Ct method (2^−ΔΔCt^). Details of the primer sequences are provided in Table 1.

### 2.12. LDH Release Assay

After the bacterial infection cell experiment, the cell culture supernatant in the 96-well plate was harvested, and the bacteria and debris were eliminated via centrifugation at 1000 rpm and filtration through a 0.22 μm membrane filter. Subsequently, in accordance with the guidelines of the LDH detection kit, the absorbance was assessed at a wavelength of 450 nm. The LDH release rate was computed to determine the extent of cellular damage using the formula:

(2) $$\mathrm{LDH}\;\mathrm{release}\;\mathrm{rate}\;=\;\frac{{OD}_{treatment\;group}\;-\;{OD}_{spontaneous\;release}}{{OD}_{maximum\;release}\;-\;{OD}_{spontaneous\;release}}\times \;\mathrm{100\%}$$

The OD of the treatment group was measured after adding MSG of various concentrations. The spontaneous release of cells occurred just with inoculation cells, and the entire media was introduced without any infection or stimulating treatment. The maximum release group of cells was supplemented with cell lysate in the cell well at the same density as the treatment group, ensuring complete cell lysis and the total release of intracellular LDH. This control denoted the theoretical maximum value of LDH within the system and was employed for standardized calculations. Additionally, an equivalent volume of complete media was provided to the control group, devoid of cell inoculation. This control was utilized to ascertain the background signal potentially produced by the medium’s components at the detection wavelength.

### 2.13. Metabolic Activity Assay

The metabolic activity of the PAO1 strain was detected and analyzed by the Alamar Blue assay. After first collecting cells from 48-well plates using fresh EP tubes, centrifuging them (5000 rpm, 5 min), washing with PBS, and resuspending in 1 mL of PBS, they were added to 96-well plates. Then 10 μL of Alamar Blue dye was added to each well and incubated at 37 °C in the dark for 1 h. A blank control group was established using PBS that contained only dyes. The absorbance at 570 nm and 600 nm was detected under a microplate reader, and the metabolic activity was calculated according to the following formula:

(3) $$\mathrm{Metabolic}\;\mathrm{activity}\;(\%)\;=\;\frac{{((E}_{oxi(OD600)}\;\times \;T_{OD570})-\;{(E}_{oxi\left(OD570\right)}\;\times \;T_{OD600}))}{{((E}_{red(OD570)}\;\times \;B_{OD600})-\;{(E}_{red(OD600)}\;\times \;B_{OD570}))}\times \;\mathrm{100\%}$$

- Eoxi (OD570)—extinction coefficient of AB in its oxidized form at 570 nm = 80,586;
- Ered (OD570)—extinction coefficient of AB in its reduced form at 570 nm = 155,677;
- Eoxi (OD600)—extinction coefficient of AB in its oxidized form at 600 nm = 117,216;
- Ered (OD600)—extinction coefficient of AB in its reduced form at 600 nm = 14,652;
- B—blank;
- T—samples.

### 2.14. ELISA Assay

First, the Tissue Homogenizer (SWE-C6, Wuhan Servicebio Technology Co., Ltd., Wuhan, China) was used to grind the lung tissue samples into lung tissue homogenates (containing 10% of the tissue weight). After centrifugation, the supernatant was then collected for related testing. Subsequently, the levels of IL-1β, MPO, SOD, and CAT in the alveolar lavage fluid and lung tissue homogenates were measured using specific commercial detection kits according to the instructions of the manufacturers. The colorimetric development was performed, and the absorbance of each well was measured at 450 nm using Multimode Plate Reader (EnSpire, PerkinElmer, Waltham, MA, USA). The concentrations of the target analytes were then calculated based on the standard curve.

### 2.15. Molecular Docking and Dynamic Simulation

The three-dimensional structures of PopB and PopD, the translocator proteins of the type III secretion system of *P. aeruginosa*, were acquired from the Protein Data Bank (PDB). The C and D chains of 4JL0 served as templates, and Discovery Studio software (v4.0) was utilized for pretreatment processes, including hydrogenation and dehydration. The CDOCKER DUCKING was then employed to dock with MSG molecules. To identify potential targets, a semi-flexible docking approach was employed to optimize the binding orientation of MSG against candidate proteins. Based on the docking results, PopB was selected for further analysis due to its favorable docking score (>50) and negative binding energy. Subsequently, molecular docking and molecular dynamics simulations were performed to characterize the MSG–PopB interaction in detail.

The molecular dynamics simulations were carried out with noncommercial Desmond/Maestro (version 2022.1) as a molecular dynamic software. TIP3P water molecules were added to the systems, which were then neutralized by 0.15 M NaCl solution. After minimization and relaxation of the system, the production simulation was performed for 100 ns in an isothermal–isobaric ensemble at 300 K and 1 bar. The trajectory coordinates were recorded every 100 ps. Molecular dynamics analysis was performed using the Simulation Interaction Diagram from Desmond.

### 2.16. SPR Assay

First, both flow cells of a CM5 sensor chip were activated with 200 μM of EDC and 50 μM of NHS (10 μL/min, 420 s) at 25 °C on a BIA core 1K system. This was followed by immobilization of the target protein (in 10 mM sodium acetate, pH 5.0) in the sample cell through two consecutive injections (50 μL, 10 μL/min, 420 s each), while the reference cell was treated with PBS (pH 5.0). Subsequently, both flow cells were blocked with 1 M ethanolamine. Following equilibration with PBS, analyte solutions at varying concentrations were injected (10 μL min^−1^, 150 s), with the surface being regenerated between cycles using 10 mM of glycine-HCl (pH 2.0). Reference-subtracted sensorgram data, collected using Biacore Insight software (v.2.0), were globally fitted to a 1:1 Langmuir binding model with the BIAcore 1K (Cytiva, Marborough, MA, USA) to obtain the kinetic constants. The final figures were prepared using Origin 7.

### 2.17. Western Blot Analysis

The total ileum tissue protein was extracted with RIPA buffer (#P3313B, Beytime, Hangzhou, China) containing 1 mM of PMSF (#ST505, Beytime, Hangzhou, China). The protein concentration of the samples was determined using the BCA kit (#P0010, Beyotime, Shanghai, China), calculated according to the absorbance value of the sample, and adjusted for uniformity of the concentration of different samples. The approximate total protein was separated by sodium dodecyl sulfate–polyacrylamide gel electrophoresis (SDS-PAGE) and transferred to a polyvinylidene fluoride (PVDF) membrane (Millipore, 0.22 μm, 0.45 μm, Millipore, Darmstadt, Germany). After blocking, the membrane was incubated in primary antibodies overnight at 4 °C. Then, after washing, the samples were incubated with secondary antibodies (#SA00001-2and#SA00001-1, Proteintech, 1:10,000, Wuhan, China). The protein bands were visualized using an ECL luminescence agent (SQ201, Yaze Biotechnology, Shanghai, China) and chemiluminescence detector (BioLight Biotechnology, Guangzhou, China).

### 2.18. Statistical Analysis

Statistical analysis and visualization were conducted with GraphPad Prism 9.0 software. All measurement data were presented as a mean ± standard error of the mean (SEM). Either one-way or two-way ANOVAs were employed for multiple group comparisons based on the experimental design, while Dunnett’s multiple comparison test was utilized for pairwise comparisons between the groups. The significance threshold was established at α = 0.05, with a difference being statistically significant when the *p*-value < 0.05.

## 3. Results and Discussion

### 3.1. Establishment of ALI Model Induced by PAO1 Infection in Mice

**Figure 1:**
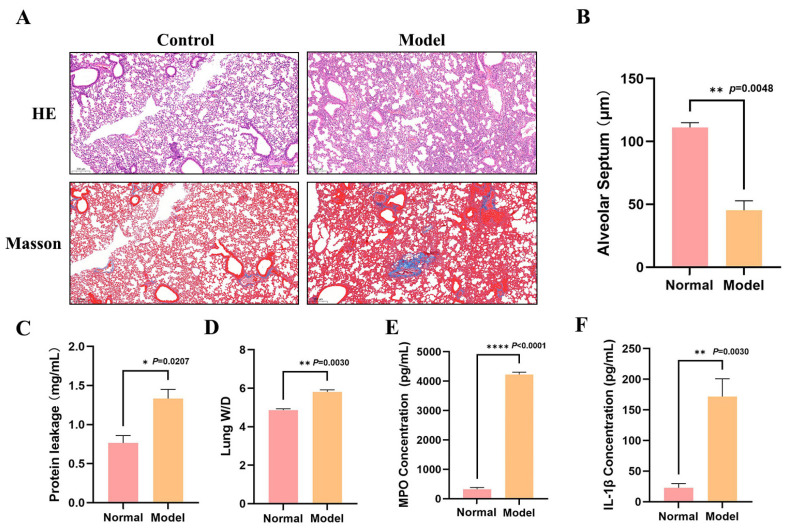
Establishment of acute lung injury models in PAO1-infected mice. (**A**) Hematoxylin and eosin (HE) and Masson’s trichrome staining of lung tissue sections; (**B**) measurement of alveolar septum distances; (**C**) protein content in bronchoalveolar lavage fluid (BALF); (**D**) lung W/D ratio in each group; (**E**) myeloperoxidase (MPO) concentration in BALF; (**F**) IL-1β concentration in BALF (n = 12 samples per group; data represent means ± SEM; * *p* < 0.05, ** *p* < 0.01, **** *p* < 0.0001).

To evaluate the pathophysiological changes underlying ALI generated by *P. aeruginosa* infection, this study effectively established an ALI model in mice based on a well-known classical approach [25]. PAO1 was injected via tracheal intubation at a dosage of 4 × 10^6^ CFU, and the lung tissue damage and inflammatory responses were measured 24 h post-infection. Our experimental results indicated that the PAO1 infection successfully generated significant ALI in mice, largely exhibiting as degradation of lung tissue architecture, increased vascular permeability, and an intense neutrophilic inflammatory response. As shown in Figure 1A,B, the PAO1 infection induced severe disruption of the mouse lung tissue architecture and considerable thickening of the alveolar septum, accompanied by extensive inflammatory cell infiltration and localized congestion, showing classic signs of pneumonic damage. Compared with the control group, the PAO1-infected group revealed significantly higher total protein levels in the bronchoalveolar lavage fluid (BALF) (Figure 1C); Moreover, the wet-to-dry weight ratio of lung tissue significantly increased (Figure 1D). Analysis of the inflammatory mediators revealed that the PAO1 infection significantly elevated the levels of proinflammatory cytokine IL-1β in BALF (Figure 1E). Additionally, MPO was greatly elevated (Figure 1F), showing substantial recruitment and activation of neutrophils at the site of infection. The results showed that in this mouse model of acute lung damage generated by *P. aeruginosa* infection, a powerful neutrophilic inflammatory response was evoked.

In studies of ALI, pulmonary edema has served as a core pathological indicator for assessing pulmonary vascular leakage and disease severity [26]. Its distinctive features physiologic indications have included a raised wet-to-dry weight ratio in lung tissue and increased protein content in bronchoalveolar lavage fluid, providing a clear indicator of the level of endothelial barrier disruption [27]. These results can collectively confirm heightened pulmonary vascular permeability and the development of pulmonary edema. Neutrophils can play a crucial role in the early stages of host defense against bacterial pathogens. Under physiological conditions, neutrophils are rarely present in healthy alveolar spaces; however, during ALI, they are rapidly recruited and can accumulate in large numbers within the pulmonary parenchyma and alveolar spaces [28]. In the host’s defense mechanisms against *P. aeruginosa* airway infections, neutrophils can play a vital role as important innate immune effector cells. The highly expressed myeloperoxidase (MPO) within these cells is a heme-dependent peroxidase that not only contributes to neutrophil antimicrobial functions but is also widely regarded as a reliable biological marker for inflammation activation and neutrophil infiltration [29,30]. In summary, tracheal injection with PAO1 can effectively establish an acute pneumonia model with typical pathological characteristics.

### 3.2. The Protective Effect of MSG on ALI Caused by PAO1 Infection in Mice

**Figure 2:**
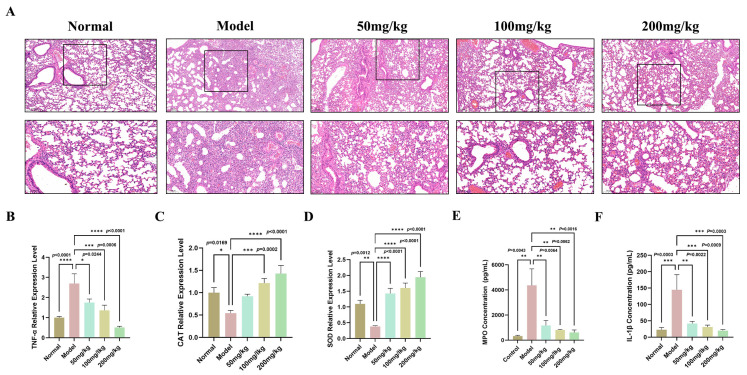
MSG alleviates *P. aeruginosa*-induced acute lung injury through reduced inflammatory infiltration, significantly narrowed alveolar septa, and well-defined alveolar structures. (**A**) HE stains results of lung tissue sections in normal, model, and different MSG dose groups; (**B**–**D**) TNF-α, CAT, and SOD mRNA transcription in lung tissues assessed using RT-qPCR; (**E**,**F**) MPO and IL-1β concentrations in bronchoalveolar lavage fluid (BALF) detected by ELISA in normal, model, and MSG-treated groups (n = 12 samples per group; data represent means ± SEM; * *p* < 0.05, ** *p* < 0.01, *** *p* < 0.001, **** *p* < 0.0001).

Following PAO1 infection, mice received intratracheal administration of different MSG doses (50, 100, and 200 mg/kg) to assess the protective effects against ALI. BALF and lung tissues were collected 24 h post-infection for subsequent analysis. HE staining revealed that, when compared with the model group infected by PAO1, mice fed with MSG exhibited alleviated lung tissue damage, reduced inflammatory infiltration, significantly narrowed alveolar septa, and well-defined alveolar structures (Figure 2A). This indicated that MSG intervention can effectively alleviate ALI induced by PAO1 infections. Subsequently, the expression levels of key inflammatory and oxidative stress-related genes in lung tissue were determined using RT-qPCR (Figure 2B–D). ELISA results indicated that MSG promoted a reduction in MPO and IL-1β levels in BALF (Figure 2E,F). MSG was shown to alleviate the inflammatory response in PAO1-infected mice, thereby exerting a protective effect on the lung tissue.

Tumor Necrosis Factor-α (TNF-α) has been a core driver in the pathogenesis of ALI, playing a pivotal role in initiating, amplifying, and sustaining the pulmonary inflammatory response [31]. TNF-α can recruit neutrophils from the bloodstream, causing them to adhere and migrate to the infected lung tissue. Neutrophil infiltration (elevated MPO levels) and inflammatory cell infiltration can be observed in histopathological sections [32,33], where it can disrupt tight junctions between cells and increase the permeability of the alveolar–capillary membrane. This can lead to the leakage of protein-rich fluids, which further exacerbates inflammation and impairs gas exchange [34]. Within the complex pathological network of ALI, oxidative stress can serve not only as a direct agent of tissue damage but also as a core amplifier driving disease progression. Its central mechanism lies in the excessive production of reactive oxygen species (ROS) and the disruption of endogenous antioxidant defense systems. Excessive ROS can lead to cellular lipid peroxidation, protein oxidation, DNA damage-induced apoptosis, activation of pro-inflammatory pathways, and increased expression of pro-inflammatory factors, thereby amplifying the inflammatory storm [35,36]. Catalase (CAT) and superoxide dismutase (SOD) can serve as the most critical endogenous antioxidant enzymes, playing an important role in countering oxidative damage and maintaining cellular homeostasis during this process. Therefore, SOD and CAT activity can directly influence the level of oxidative stress, as its downregulation in expression or function can lead to a sudden surge in oxidative stress [37,38]. Previous studies have demonstrated that MSG combined with L-arginine can enhance the capacity to resist oxidative stress [24]. A similar phenomenon was observed in this experiment, as MSG treatment significantly upregulated the gene expression of CAT and SOD (Figure 2C,D). These findings indicated that MSG not only alleviated inflammatory damage but may have also enhanced the antioxidant defense capacity of lung tissue. It maintained intracellular antioxidant balance by counteracting the infection-induced oxidative stress, which protected the structure and function of the lung tissue.

### 3.3. MSG Reshapes Macrophage Polarization in the Mouse ALI Model

**Figure 3:**
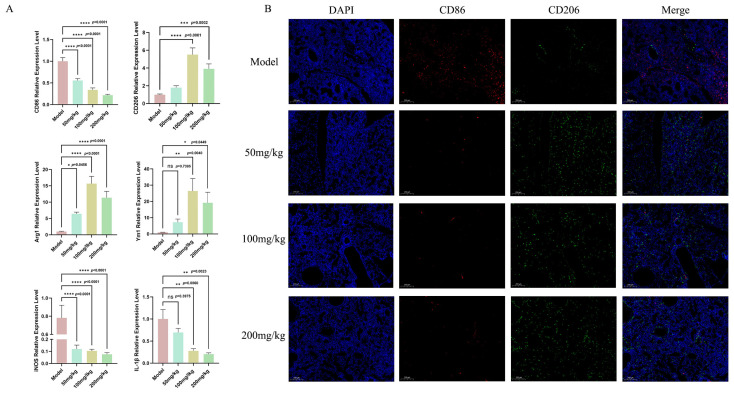
MSG inhibits polarization of M1 macrophages and facilitates polarization of M2 macrophages in lung tissue during *P. aeruginosa* infection. (**A**) CD86, CD206, Arg1, Ym1, iNOS, and IL-1β mRNA transcription levels in lung tissues in model and MSG-treated groups (n = 12 samples per group; data represent means ± SEM; * *p* < 0.05, ** *p* < 0.01, *** *p* < 0.001, **** *p* < 0.0001, ns: no significant difference.); (**B**) detection of CD86 and CD206 protein expression in lung tissues using immunofluorescence in model and the MSG-treated groups.

Macrophages are a group of cells that can detect infections, such as bacteria or pathogens, and initiate the initial immune response. Additionally, alveolar macrophages can serve as the primary source of TNF-α in the early stages of pneumonia [39]. Macrophage polarization toward the M1 (pro-inflammatory) or M2 (anti-inflammatory) phenotypes represents a critical immunomodulatory mechanism that can govern the dynamics of inflammatory progression and resolution [40,41]. Therefore, when exploring the regulatory mechanisms of pulmonary inflammation, the polarization state of macrophages has been considered a key immune behavior determining the course and outcome of inflammation. Among these, classically activated M1 macrophages can highly express marker molecules such as iNOS, CD86, and IL-1β, primarily exerting pro-inflammatory and pathogen clearance functions [42,43]. In contrast, alternatively activated M2 macrophages can highly express Arg1, Ym1, and CD206, participating in inflammation resolution, tissue repair, and immune regulation [14,41,44]. In acute lung injuries, the dynamic balance between these two states can profoundly influence disease progression and outcomes. Based on this, the present study further investigated the effects of MSG treatment on macrophage polarization. In this study, MSG administration dramatically lowered mRNA expression levels of M1 macrophage phenotypic markers iNOS, CD86, and IL-1β compared to the model group. Concurrently, mRNA expression levels of the M2 macrophage phenotype markers Arg1, Ym1, and CD206 were significantly higher compared to the model group (Figure 3A). Immunofluorescence staining of the lung tissue using CD86 and CD206 markers for the M1 and M2 macrophages revealed a reduced M1/M2 ratio (Figure 3B). These findings demonstrated that, in the PAO1-induced acute lung injury mouse model, MSG treatment alleviated lung tissue damage and inflammation by promoting macrophage M2 polarization and reducing the M1/M2 ratio.

Integrating previous data, these results could suggest that the regulatory function of MSG is directly linked to its ability to alter the redox balance within the lungs. It is known that M1 macrophage activation and mitochondrial ROS bursts are causally interdependent [45]. MSG may break the positive feedback loop of M1 polarization by enhancing endogenous antioxidant defenses such as SOD and CAT to scavenge excess ROS. Simultaneously, the alleviation of oxidative stress may have directly or indirectly promoted the phenotypic shift in macrophages toward the M2 phenotype. Hence, macrophage polarization could be postulated as a central integrative mechanism through which MSG can exert its concomitant antioxidant and anti-inflammatory effects.

### 3.4. MSG Alleviates Inflammatory Responses in a PAO1-Induced Mouse ALI Model via the TLR-4/MAPK Pathway

**Figure 4:**
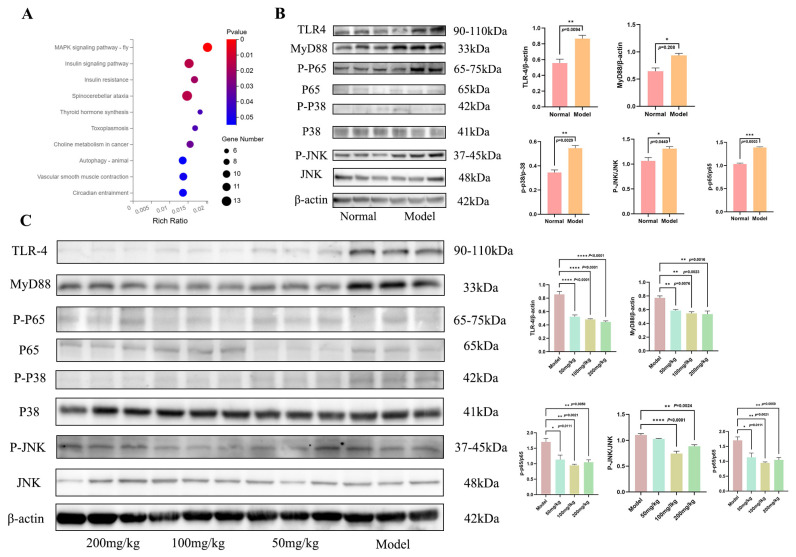
KEGG pathway enrichment analysis and key protein expression in *P. aeruginosa*-induced acute lung injury (ALI) in mice. (**A**) KEGG pathway enrichment of differentially expressed genes between the ALI model and control groups; (**B**) protein levels of phospho-JNK, p-p38, p-p65, TLR4, and MyD88 in lung tissues compared between the ALI model and control groups by Western blot analysis (β-actin loading control); (**C**) comparison of protein levels (p-JNK, p-p38, p-p65, TLR4, MyD88) in lung tissues between the model and MSG-treated groups, as detected by Western blot (β-actin control) (n = 12 samples per group; data represent means ± SEM; * *p* < 0.05, ** *p* < 0.01, *** *p* < 0.001, **** *p* < 0.0001).

Based on transcriptomic analysis of PAO1 infection-induced acute lung injury in mice, the MAPK pathway was significantly enriched after infection (Figure 4A). Subsequent Western blot analysis confirmed that the infection significantly activated the upstream key adaptor protein MyD88 and its associated pattern recognition receptor TLR4 in this pathway, while substantially elevating the phosphorylation levels of downstream core kinases p38 and JNK (Figure 4B). Following MSG intervention, the activation status of the key signaling molecules was significantly suppressed (Figure 4C). By combining this series of data, this demonstrated that MSG could take advantage of its lung-protective benefits by suppressing the TLR4/MyD88/MAPK signaling axis to prevent excessive inflammatory responses.

The excessive activation of the TLR4/MyD88/MAPK axis acted as the central signaling driver of the inflammatory storm in infection-induced ALI. Specifically, pathogen-associated molecular patterns (PAMPs) from *P. aeruginosa* were recognized by TLR4 on innate immune cells, including alveolar macrophages. This recognition recruited the adaptor protein MyD88, thereby initiating downstream signaling cascades. In this study, the findings of TLR4 and MyD88 upregulation, alongside enhanced phosphorylation of p38 and JNK in the model group, has provided direct evidence of this pathway being strongly activated by pathogens. This can also perfectly explain the phenotypes observed in earlier studies, including the explosive release of cytokines such as TNF-α and the subsequent intensity of neutrophil infiltration. Moreover, the inhibition of this pathway by MSG functioned as an upstream switch, accounting for its multifaceted protective effects which reduced pro-inflammatory factor levels, alleviated oxidative stress, and promoted macrophage polarization toward the reparative M2 phenotype. The findings could possibly provide a unified upstream explanation for these phenotypes: MSG severed excessive inflammatory signaling by inhibiting the TLR4/MyD88/MAPK axis at its source. Thus, this regulation could represent a convergent upstream mechanism, offering a coordinated basis for its triple-action protective effects encompassing anti-inflammatory, antioxidant, and immunomodulatory activities.

### 3.5. The Inhibitory Effect of MSG on PAO1 In Vitro Infection with Macrophage

**Figure 5:**
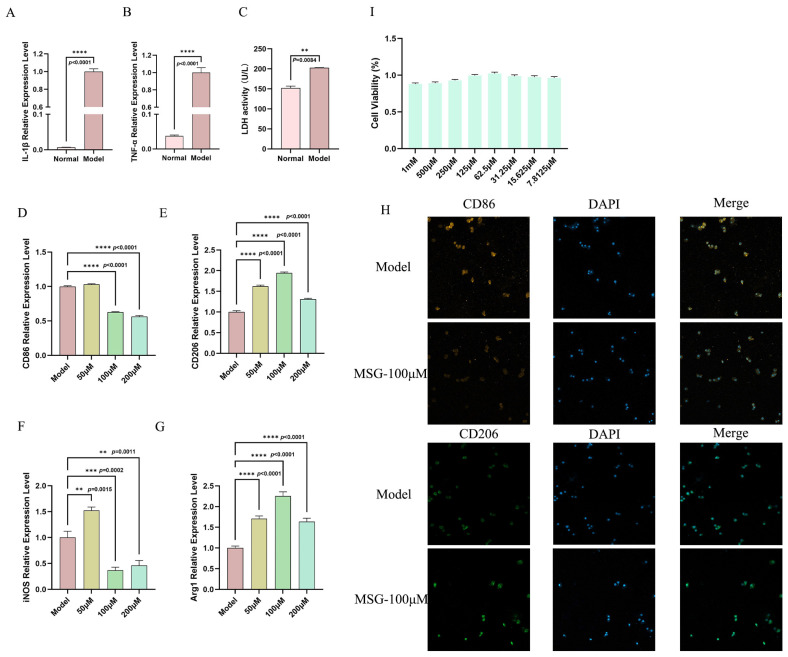
MSG treatment shifts the balance of macrophage polarization from a pro-inflammatory M1 phenotype to an anti-inflammatory M2 phenotype. (**A**,**B**) The levels of IL-1β, TNF-α, and LDH release in macrophages after PAO1 infection; (**C**) the release of LDH in macrophages after PAO1 infection; (**D**–**G**) the mRNA transcription of CD86, CD206, iNOS, and Arg1 in macrophages after the treatment of MSG at different concentrations (n = six samples per group; data represent means ± SEM; ** *p* < 0.01, *** *p* < 0.001, **** *p* < 0.0001); (**H**) immunofluorescence analysis of CD86 and CD206 protein levels in macrophages from the infection model and control groups; (**I**) CCK-8 assay of macrophage viability with MSG.

The results from the previous experiments demonstrated the intense inflammatory response triggered by the infection. Therefore, we further investigated the role of MSG in an in vitro infection model. First, we established an MH-S cell infection model in vitro. Compared with the control group, the expression levels of IL-1β and TNF-α as well as the release level of LDH in the model group were significantly upregulated (Figure 5A–C). Following MSG intervention, these signals significantly receded. Simultaneously, the expression of CD206 showed a marked increase while CD86 showed a reduction following MSG treatment (Figure 5D-E). The expression levels of iNOS and Arg1 mRNA in the macrophages exhibited the same phenomenon observed in the tissue: an increased Arg1 gene expression accompanied by a decreased iNOS gene expression (Figure 5F–G). Moreover, as clearly demonstrated by immunofluorescence images (Figure 5H), intense CD86 fluorescence signals were widely distributed in a speckled pattern across the cell surface in the model group, highly overlapping with dense DAPI nuclear signals. This pattern aligned with the characteristic high expression of co-stimulatory molecules in activated M1-type macrophages. Following treatment with 100 μM of MSG, the fluorescence intensity was markedly reduced. In stark contrast, the CD206 signal in the model group not only exhibited significantly enhanced fluorescence intensity after MSG treatment but also displayed a more diffuse and uniform distribution pattern. These studies demonstrated high consistency with the TLR4/MyD88/MAPK signaling pathway, which can serve as a key upstream signal initiating M1 polarization and pro-inflammatory cytokine production. This delineated a clear signaling pathway: MSG suppressed TLR4/MAPK pathway activation, thereby reducing proinflammatory gene transcription, altering macrophage polarization, and ultimately mitigating inflammation and tissue damage.

### 3.6. MSG Combined with PopB for Molecular Docking and Kinetic Simulations

**Figure 6:**
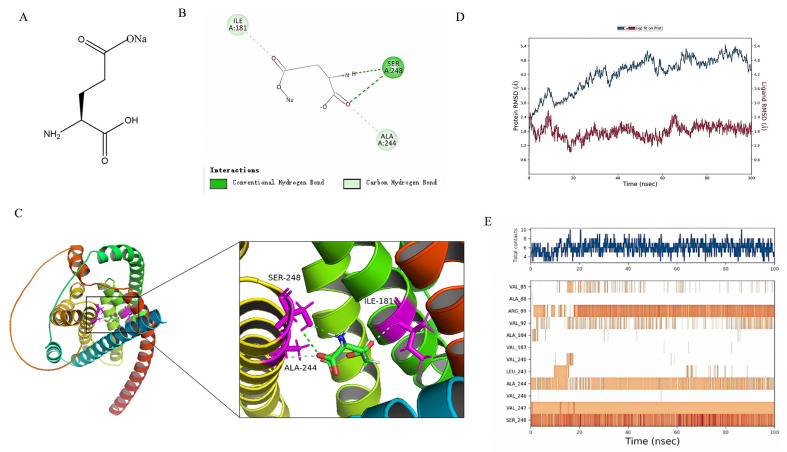
Molecular docking reveals the binding mode of MSG within the active site of PopB protein. (**A**) Two-dimensional chemical structure of MSG. (**B**) Three-dimensional binding pose of MSG within the active site of PopB protein. (**C**) Location of key interacting residues (highlighted in red) on the PopB protein structure. (**D**) Backbone root-mean-square deviation (RMSD) of the PopB protein in complex with MSG during a 100 ns molecular dynamics simulation. (**E**) Per-residue root-mean-square fluctuation (RMSF) of the PopB protein in complex with MSG.

The protective role of MSG and its underlying molecular mechanisms in the host during *P. aeruginosa*-induced acute lung injury in mice have been previously explored; however, whether MSG can also directly influence the pathogen itself remains to be investigated. The T3SS of *P. aeruginosa* served as a critical virulence apparatus essential for its pathogenicity. Given its pivotal role in initiating acute infections, particularly in the context of pulmonary acute infection where it can mediate effector injection into host cells, T3SS was selected as a strategic target for molecular docking studies [46,47]. The results indicated that MSG bound to PopB in an optimal conformation with a binding energy of −19.179 kcal/mol (Figure 6B). Multiple interactions formed during the docking process between this small molecule and the PopB protein, with hydrogen bonds serving as the primary force. The SER248 amino acid played a crucial role in this process.

To assess the stability of the MSG–PopB complex, the binding interaction was analyzed over a 100 nanosecond molecular dynamics simulation. Molecular dynamics simulations revealed that the root-mean-square deviation (RMSD) of the protein scaffold in the complex system reached equilibrium within the range of 4.2–5.4 Å, while the RMSD of the MSG ligand ultimately stabilized at approximately 1.8 Å (Figure 6C). This data indicated that during the simulation period, both the overall conformation of the PopB protein and the position of MSG within its binding pocket stabilized without significant drift, suggesting the formation of a stable binding conformation between the two. As a small-molecule ligand, MSG’s low RMSD value particularly indicated its highly stable orientation and position within the binding pocket, forming a crucial structural basis for its effective biological interactions. Further analysis of the binding mode dynamics revealed key molecular forces underpinning stable binding. Throughout the 100 ns simulation, the MSG–PopB binding interface was maintained by multiple noncovalent forces, including hydrogen bonds, hydrophobic interactions, and electrostatic interactions (Figure 1C). Notably, serine residue SER248 at position 248 of the PopB protein maintained a highly stable and persistent interaction (e.g., hydrogen bond formation) with MSG throughout the simulation. SER248 likely functioned as a critical anchor residue for MSG–PopB binding. The polar hydroxyl group of its side chain appeared to directly engage specific functional groups of MSG, thereby playing a decisive role in stabilizing the overall conformation of the complex.

Molecular docking and molecular dynamics simulation analysis of MSG and PopB protein led to the proposal that MSG can directly target and stably bind to the PopB protein. This binding could likely disrupt its function through two distinct mechanisms: first, via steric hindrance that directly obstructs the formation of the translocation channel or the transit of effector proteins; second, through allosteric modulation, whereby MSG binding induces subtle conformational changes in PopB, impairing its assembly with partner proteins (e.g., PopD, PcrV) or its efficiency of membrane insertion into host cells [48]. Regardless of the mechanism of functional disruption, both pathways could inhibit PAO1 infection of host cells, potentially reducing inflammation and tissue damage.

### 3.7. SPR

**Figure 7:**
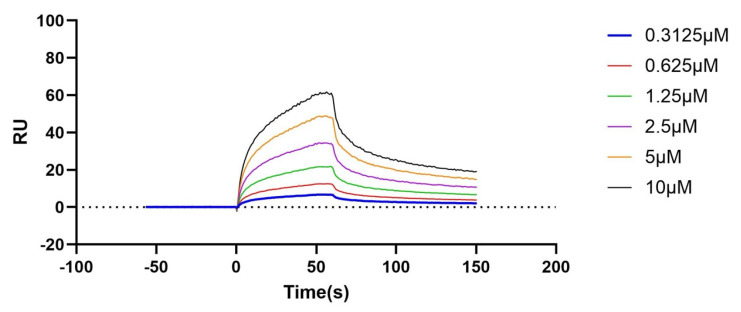
Affinity analysis diagram of the interaction between the PopB protein and MSG.

Based on the molecular docking and kinetic simulation predictions, MSG may occupy the functional interface or influence the conformation of PopB protein by forming stable hydrogen bonds with residues such as SER248. However, the strong binding affinity and stable conformation suggested by the computational simulations would require direct experimental validation. SPR technology was employed to experimentally verify the direct interaction between MSG and PopB protein in vitro, enabling quantitative and real-time determination of their binding affinity (*K*_D_ value). A carboxymethylated dextran sensor chip (CM 5 series), functionalized with the target protein, was used to determine the binding affinity. The measured dissociation constant (*K*_D_) between the compound and PopB was 3.52 × 10^−6^ M. The dissociation constant (*K*_D_) reflected the affinity of the analyte for the target, where a smaller value can indicate stronger affinity. The results demonstrated that MSG exhibited strong binding affinity with PopB. As the MSG concentration increased from 0.3125 μM to 10 μM, both the binding response values and the final equilibrium plateau values on the curve systematically and significantly increased (Figure 7). This observation demonstrated a typical, specific binding process between MSG and the immobilized target that was concentration dependent, in which more binding sites can become occupied with an increasing analyte concentration until a new binding-dissociation equilibrium is established at each concentration.

This SPR experiment confirmed the prediction from the molecular dynamics simulations that MSG can directly and specifically bind to PopB, the translocator protein of the *P. aeruginosa* T3SS, with a binding affinity at a significant sub-micromolar level.

### 3.8. MSG Inhibits the Translocation of PAO1 Effector Proteins into MLE-12/CHO-K1 Cells In Vitro

**Figure 8:**
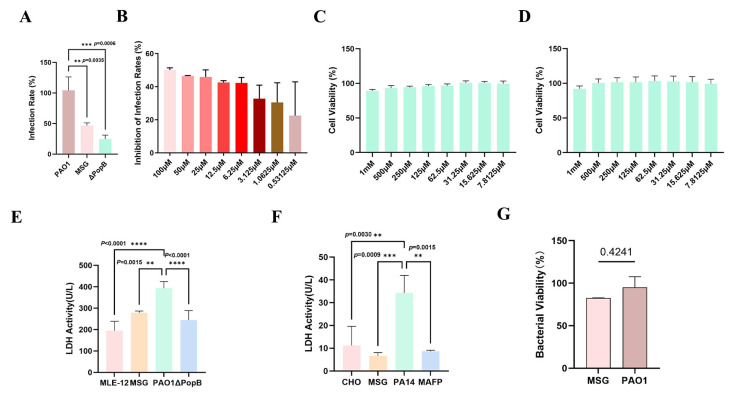
Inhibitory effects of MSG on the type III secretion system (T3SS) of *Pseudomonas aeruginosa*. (**A**) Infection rates of MLE-12 cells challenged with PAO1 (PAO1-exoT-bla) in the presence or absence of 100 μM of MSG, or with the PopB mutant (ΔPopB), quantified by the CCF4-AM assay; (**B**) inhibition of infection quantified by the CCF4-AM assay following challenge with PAO1 (PAO1-EXOT-bla) in the presence of MSG at different concentrations; (**C**,**D**) CCK-8 assay of MLE-12 and CHO-K1 cell viability in the presence of MSG; (**E**) LDH release measured over a 3 h infection period in MLE-12 cells challenged with PAO1 (PAO1-EXOT-bla) in the presence or absence of 100 μM of MSG, or with the ΔPopB; (**F**) LDH release measured over a 1 h infection period in CHO-K1 cells challenged with PAO1 (PAO1-EXOT-bla) in the presence or absence of 100 μM of MSG, or with the ΔPopB; (**G**) bacterial viability after treatment with MSG (n = six samples per group; data represent means ± SEM; ** *p* < 0.01, *** *p* < 0.001, **** *p* < 0.0001).

Following the validation of the molecular interaction, bacterial infection models were established using two distinct animal cell lines—the mouse lung epithelial cell line MLE-12 and the Chinese hamster ovary cell line CHO-K1—to further confirm the inhibitory effect of MSG on the *P. aeruginosa* T3SS. Subsequently, the suppression of bacterial virulence functions by MSG was assessed by measuring T3SS-dependent cytotoxicity and LDH release. CCK-8 assays revealed no significant toxicity of MSG at concentrations up to 1 mM in both MLE-12 and CHO-K1 cells (Figure 8C,D). Therefore, concentrations within the non-toxic range were selected for all subsequent functional assays.

First, using the PME-exoT-bla PAO1 strain constructed in our laboratory, the cell lines were infected. Fluorescence Resonance Energy Transfer (FRET) technology was employed to detect the fluorescence intensity of CCF4-AM, and CCF4 was used as a designed substrate probe based on the FRET principle. Studies have shown that MSG can effectively reduce effector translocation rate at a concentration of 100 μM, achieving an inhibition efficiency of up to 50% (Figure 8A). Subsequently, the effect of concentration on its inhibitory efficacy was examined. The compound exhibited a consistent inhibition rate exceeding 42% across the concentration range of 6.25 to 100 μM (Figure 8B). This demonstrated that MSG exhibited significant and stable inhibitory effects against bacterial virulence within the micromolar concentration range. To further assess its cellular compatibility and ability to mitigate infection-induced damage, MSG was subjected to a series of functional assays. At the effective concentration of 100 μM, MSG significantly reduced LDH release following infection with PAO1 and PA14 strains, indicating its ability to mitigate infection-induced cell damage (Figure 8E,F). Furthermore, through the metabolic activity test of bacteria, it was found that MSG did not affect the metabolic activity of bacteria (Figure 8G).

This study established a cell line infection model and combined it with FRET detection technology based on the ExoT-β-lactamase reporter gene. The experimental results demonstrated that MSG effectively disrupted translocation of the effectors of T3SS, exhibiting significant efficacy across a broad concentration range. Notably, at the effective concentration of 100 μM, MSG significantly attenuated LDH release induced by PAO1 and PA14 infection without interfering with bacterial metabolic activity.

## 4. Conclusions

Studies have demonstrated that MSG can exert an inhibitory effect on effector translocation by binding to the PopB protein of T3SS in *Pseudomonas aeruginosa*, thereby mitigating the cellular damage induced by acute *P. aeruginosa* infection. Additionally, MSG can regulate macrophage polarization and excessive inflammatory responses associated with *P. aeruginosa*-induced acute lung injury through the inhibition of the TLR-4/MyD88/MAPK signaling pathway. These findings can provide a novel therapeutic strategy for combating bacterial infections and establish a foundational basis for the development of bifunctional drugs targeting host–pathogen interactions.

## Figures and Tables

**Table 1:** The primer sequences of experimental target genes.

Target Genes		Sequences (5′-3′)
Arg1	Forward	AAGACAGCAGAGGAGGTGAAGAG
	Reverse	GGTAGTCAGTCCCTGGCTTATGG
CD206	Forward	TGCCACTGCCATGCCTACC
	Reverse	GCTTGCCGTGCGTCTTGC
Ym1	Forward	CCACAGGAGCAGGAATCATTGAC
	Reverse	TTCTCCAGTGTAGCCATCCTTAGG
CD86	Forward	AGCACTATTTGGGCACAGAGAAAC
	Reverse	GAAGTCGTAGAGTCCAGTTGTTCC
iNOS	Forward	TCACTCAGCCAAGCCCTCAC
	Reverse	TCCAATCTCTGCCTATCCGTCTC
IL-1β	Forward	TCGCAGCAGCACATCAACAAG
	Reverse	TCCACGGGAAAGACACAGGTAG
TNF-α	Forward	ACGTGGAACTGGCAGAAGAGG
	Reverse	TGAGAAGAGGCTGAGACATAGGC
CAT	Forward	CGGCACATGAATGGCTATGGATC
	Reverse	TGGTCGGTCTTGTAATGGAACTTG
SOD	Forward	GCACCACAGCAAGCACCAC
	Reverse	CTGAAGAGCGACCTGAGTTGTAAC

## Data Availability

The original contributions presented in this study are included in the article. Further inquiries can be directed to the corresponding authors.
